# Study on the influence of new quality productivity on carbon emissions of the manufacturing industry

**DOI:** 10.1038/s41598-025-10827-z

**Published:** 2025-07-18

**Authors:** Ding Han, Panlong Sheng, Rishuai Xing, Fuxiang Xu

**Affiliations:** 1 School of Business Administration, Liaoning Technical University, Huludao, China; 2 School of Public Administration, Shandong Technology And Business University, Yantai, 264000 China

**Keywords:** New quality productivity, Manufacturing carbon emissions, Labour productivity in manufacturing, Industrial agglomeration, Environmental social sciences, Environmental economics, Sustainability

## Abstract

Using 30 provinces, autonomous regions, and municipalities in China (excluding Tibet, Hong Kong, Macau, and Taiwan) as research samples from 2012 to 2022, the study examines the nonlinear impact of new quality productivity on carbon emissions in the manufacturing industry. It discusses the mediating role played by labour productivity in manufacturing and industrial agglomeration between the two. The study finds that: there is an inverted U-shaped relationship between new productivity and manufacturing carbon emissions; labour productivity in manufacturing and industrial agglomeration play a partly intermediary role in the inverted U-shaped relationship, with the most substantial intermediary effect played by industrial agglomeration, followed by labour productivity in manufacturing; the test of heterogeneity shows that: compared with the economically underdeveloped and low-urbanized provinces and municipalities, the impacts of new productivity of the economically developed and highly urbanized provinces and municipalities on manufacturing carbon emissions show a significant inverted U-shaped relationship, followed by a strong intermediary effect played by labour productivity in manufacturing. The heterogeneity test shows that compared with the economically less developed and low urbanization level provinces and cities, the new quality productivity of economically developed and high urbanization level provinces and cities shows a significant inverted U-shaped relationship on carbon emission in the manufacturing industry.

## Introduction

With the global response to climate change and the landing of China’s “double carbon” goal, the green development of the manufacturing industry has become the background colour of its high-quality development. However, the current proportion of carbon emissions in China’s manufacturing industry is still high^1^. The high emissions and high energy consumption of the manufacturing industry have formed resistance to achieving the “dual carbon” goal. Productive forces are the fundamental force for promoting high-quality and green economic and social development, but the traditional productive forces with extensive capital expansion as the main development mode make it difficult to achieve the green and sustainable development goals of economic and social development. The proposal of the new quality productive forces brings a new opportunity for the low-carbon development of our country’s economic society. Under the background of green economic transformation, all industries are actively exploring new low-carbon development paths. As an important link of economic development, can the manufacturing industry achieve the goal of reducing carbon emissions with the help of new quality productivity in practice?

Qualitative studies on the effect of new quality productivity on carbon reduction have been abundant. According to existing studies, the new quality productivity is a green productivity with a prospect of sustainable development^2^. Through the rapid evolution and deep integration of digital technologies, it promotes the transformation of the economy and society into an intelligent and networked dimension, to fully release the green energy that meets the requirements of sustainable development^3^. However, existing studies have not quantitatively analyzed the relationship between new quality productivity and carbon emissions, nor have they conducted in-depth studies on the mechanism of new quality productivity on carbon emissions in the manufacturing industry, which makes it difficult to provide references for the manufacturing industry to accurately locate the key application points of new quality productivity in carbon emission reduction in the manufacturing industry^4–7^. And then form a dependence on this high-carbon mode, ultimately making the manufacturing industry prone to a high-carbon locking effect.

New quality productivity represents advanced production technologies and organizational methods, which show great potential in promoting economic restructuring and green development^8^. New quality productivity advocates enhancing labour productivity in manufacturing^9^ and industrial agglomeration^10^. The core of new quality productivity lies in the use of disruptive and cutting-edge technologies as the engine. At the level of labour productivity, the new quality productivity realizes real-time perception, dynamic optimization and precise control of the production process through intelligent manufacturing, flexible automation and digital twin systems, significantly compresses ineffective working hours, optimizes resource allocation, and relies on big data and machine learning to improve the decision-making efficiency of the complex process, to make the output of the unit of labour input continue to jump up. At the level of industrial agglomeration, the new quality of productivity creates a reliance on highly specialized support systems and knowledge-intensive service ecosystems, prompting the accelerated convergence of innovation factors to specific regions.

Improving labour productivity and industrial agglomeration in the manufacturing industry can have an impact on carbon emissions in many ways. On the one hand, through technological innovation and process optimization, energy consumption per unit of product is reduced and energy use efficiency is improved, which directly reduces carbon emissions in the production process^11^; On the other hand, efficient production accelerates industrial upgrading and promotes the application of green technologies to curb the growth of carbon emissions at the source^12^. Similarly, industrial agglomeration can also play a role in carbon emissions, which mainly lies in the scale effect, technology and knowledge spillover effect and industrial structure optimization effect^13^. A large number of enterprises in a particular region, large-scale energy procurement and supply to improve energy efficiency, but also conducive to the centralized treatment of waste, reduce energy consumption and emissions; and industrial agglomeration can also promote the synergistic development of upstream and downstream industries, promote the optimization and upgrading of industrial structure, eliminating the backward production capacity of high energy-consuming^14^. Therefore, this paper incorporates new quality productivity, manufacturing carbon emissions, labour productivity in manufacturing, and industrial agglomeration into the same framework, based on data from 30 provinces, autonomous regions and municipalities in China (excluding Tibet, Hong Kong, Macao, Taiwan), to explore the impact of new quality productivity on manufacturing carbon emissions as well as the intermediary roles of labour productivity in manufacturing and industrial agglomeration in it.

Given this, the marginal contributions of this paper are: firstly, it expands the realization path of mitigating carbon emissions in the manufacturing industry. Based on the Marxist productivity theory, this paper innovatively quantitatively examines the association between new quality productivity and manufacturing carbon emissions from a non-linear perspective, verifies the inverted "U" relationship between the two, and provides a new research idea for carbon emission reduction in the manufacturing industry. Secondly, it reveals the conduction path of the nonlinear influence of new productivity on carbon emissions in the manufacturing industry. Based on the theory of classical economics and the theory of externalities, labour productivity in manufacturing and industrial agglomeration are introduced into the nonlinear impact of new productivity on carbon emissions in the manufacturing industry, and their intermediary role in the nonlinear relationship is examined, which clarifies the internal mechanism of the relationship between new productivity and carbon emissions in the manufacturing industry, and deepens the understanding of the important role of labour productivity in manufacturing and industrial agglomeration.

## Theoretical analysis and research hypothesis

### New quality productivity and carbon emissions of the manufacturing industry

Marxist productivity theory is the most fundamental theoretical cornerstone of Marxist theory, including “labour productivity”, “material productivity and spiritual productivity”, “natural productivity”, “social productivity”, “industrial productivity”, “modern productivity” and other conceptual categories^15^, including a clear ecological dimension. It can provide theoretical support for the green development of new quality productivity^16^. The cultivation and development of new quality productivity is a long-term task and system engineering in China, which takes green development as the basic direction and factor allocation as the key means, can have a great influence on the realization of the sustainable development goal of the manufacturing industry. In the long run, with the development level of new quality productivity reaching a certain level, carbon emissions from manufacturing will be suppressed. After long-term cultivation and development, the new quality productivity will produce a certain scale effect^17^, which can lead to the green development direction of the manufacturing industry. By promoting the manufacturing industry to strengthen the use of new materials and new energy, the new quality productivity helps the green transformation and green technology innovation of the manufacturing industry, reduces the energy consumption per unit output value of the manufacturing industry, improves energy utilization efficiency and optimizes the industrial structure, and ultimately reduces the carbon emissions of the manufacturing industry. But in the short term, in the early stage of the development of new quality productivity, it will increase the carbon emissions of the manufacturing industry. China’s electricity output is still relatively dependent on coal, and the improvement of new quality productivity mainly relies on advanced technologies such as artificial intelligence, big data, and the Internet of Things. The application of advanced technologies requires high power consumption^18^, which will lead to an increase in carbon emissions. Therefore, this paper proposes:

Hypothesis H1: There is an inverted U-shaped relationship between new quality productivity and carbon emissions of the manufacturing industry.

### The effect of new quality productivity on carbon emissions of the manufacturing industry

#### Mediating effects of labour productivity in manufacturing

Labor productivity in manufacturing refers to the change in manufacturing labor productivity and unit labor costs labor productivity is the ratio of output to the corresponding labor consumption over a given period of time^19^. Classical economics emphasizes that the optimal allocation of factors of production, such as labour and capital, can improve production efficiency, and the increase in labour productivity means an increase in output per unit of labour input, and under the combined effect of technological progress and economies of scale, this process can achieve the optimization of resource use efficiency^20^. When labour productivity in the manufacturing industry increases, on the one hand, enterprises can adopt more efficient production equipment and processes through technological innovation and management optimization, reducing energy consumption and waste emissions per unit of product production process^21^; On the other hand, the increase in labor productivity is often accompanied by the upgrading of the industrial structure to high value-added segments, prompting enterprises to shift from relying on high-energy-consuming and high-polluting production modes to cleaner, low-carbon production methods^22^. This shift is consistent with the equilibrium theory of factor inputs and outputs in classical economics, that is, the optimal allocation of resources is realized through the improvement of factor efficiency, thus reducing the negative impact of production activities on the environment. Therefore, in the above process, labour productivity in manufacturing will reduce carbon emissions in China’s manufacturing industry.

As an advanced productivity form with innovation as the core driving force and high-tech elements as the support, the new quality productivity is closely linked to the labour productivity of the manufacturing industry. In the early stage of the development of new quality productivity, enterprises need to invest a lot of resources in the research and development of new technologies, equipment updating and personnel training, due to the technology not yet mature, the production process is in the state of debugging and optimization, the alternation of new and old technologies may lead to a decline in the efficiency of the production system^23^. At the same time, there is a time lag for employees to adapt to new production models and technological tools, and the skills mismatch problem can reduce the efficiency of production collaboration^24^. Coupled with the high cost of initial technology application, the scale effect has not yet appeared, the superposition of multiple factors makes the new quality productivity inhibit the improvement of labour productivity in the manufacturing industry, and may even lead to the decline of labour productivity in the manufacturing industry. In the late stage of the development of new quality productivity, the development of new quality productivity into a deepening stage, technology iteration and factor adaptation mechanism gradually improved. On the one hand, the stability and reliability of the new technology have been significantly improved, and the deep integration of intelligent and digital equipment with the production system has enabled the precise allocation of resources and automated optimization of processes, which has significantly increased the output per unit of labour; on the other hand, the skill level of labourers is highly compatible with the application scenarios of the new technology through continuous learning and practice, and the efficiency of human–machine collaboration has been enhanced^25^. In addition, the industrial ecology driven by the new quality of productivity continues to mature, the upstream and downstream of the industrial chain synergistic innovation effect is highlighted, and the advantages of economy of scale and economy of scope are gradually released, prompting the manufacturing industry to achieve leapfrog growth in labour productivity.

As a result, this paper proposes:

Hypothesis H2: Labor productivity in manufacturing plays a mediating role between new quality productivity and manufacturing carbon emissions.

#### Mediating effects of industrial agglomeration

Industrial agglomeration refers to the process in which the same industry is highly concentrated in a particular geographical location and the industrial capital elements are constantly converging in the spatial scope^26^. According to the externality theory, industrial agglomeration has a significant effect on suppressing carbon emissions^27^. In industrial agglomeration areas, manufacturing industries are concentrated in similar geographic locations and share infrastructure, which avoids duplicated construction and resource wastage under a decentralized layout, improves infrastructure utilization efficiency^28^, and reduces energy consumption and carbon emissions. For example, centralized heating and power supply in industrial parks reduces energy loss and greenhouse gas emissions. In addition, industrial agglomeration also promotes the exchange of knowledge and technology within the manufacturing industry, accelerating the dissemination and application of low-carbon technologies^29^. The manufacturing industry competes and cooperates in the agglomeration, and to reduce costs and improve competitiveness, it is more motivated to research develop and adopt low-carbon technologies; meanwhile, technological spillovers enable the rapid diffusion of advanced low-carbon technologies, leading to a reduction in carbon emissions from the manufacturing industry in the whole region.

Similarly, industrial agglomeration may be affected differently at different periods of the development of new quality productivity. In the early stage of the development of new quality productivity, which is often characterized by the initial exploration of emerging technologies and innovative ideas, uncertainty and high risk can inhibit industrial agglomeration^30^. On the one hand, the immaturity of the relevant technologies and models promoted by the new quality productivity makes it difficult for the manufacturing industry to accurately assess the market outlook and return on investment, and it dares not hastily concentrate on the layout for fear of wasting resources and operational failure. On the other hand, the supporting infrastructure and institutional environment are not yet perfect, lacking the attraction for manufacturing enterprises to cluster^31^. Manufacturing industry within their decentralized research, it is difficult to form a clustering effect. However, the development of new quality productivity for some time will turn to promote industrial agglomeration. With the gradual maturation of technology and clear market prospects, the manufacturing industry sees great potential for development and has been moving closer to regions with technological advantages and an innovative atmosphere^32^. At this time, the specialized division of labour within the region is constantly refined, and the upstream and downstream enterprises within the manufacturing industry work closely together to reduce transaction costs and thus form a complete industrial chain. At the same time, the government and society will also increase the investment in related infrastructure and public services, further attracting manufacturing talents and capital agglomeration, forming a virtuous circle, promoting the accelerated development of industrial agglomeration, and constructing competitive industrial clusters.

As a result, this paper proposes:

Hypothesis H3: Industrial agglomeration plays a mediating role between new quality productivity and manufacturing carbon emissions.

**Fig. 1:**
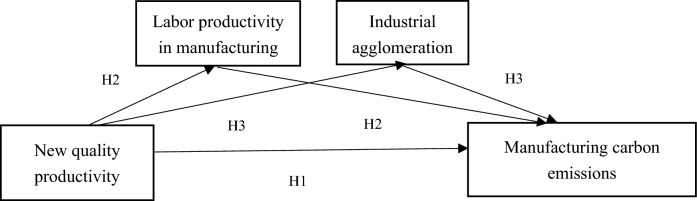
Impact mechanism pathway architecture diagram.

In summary, the mechanism of the impact of new quality productivity on carbon emissions in the manufacturing industry is summarized in Fig. 1 below.

## Model construction, variable selection and sample description

### Model construction

#### Reference regression model

To test hypothesis H1, a model is constructed as follows:

1 $$MCE_{it} = \, \beta_{0} + \, \beta_{{1}} New_{it} + \, \beta 2New_{it}^{{2}} + \, jControls_{it} + \, \tau_{i} + \, \mu_{t} + \, \varepsilon_{it}$$

where *MCE*_*it*_ is the total carbon emission, *i* represents the province, *t* represents the year, *New*_*it*_ is the core explanatory variable of this paper, representing the level of new quality productivity in each province, the explained variable *MCE*_*it*_ is the total carbon emissions of the manufacturing industry in each province, *α*_0,_ *α*_1,_ *β*_0,_ *β*_1,_ *β*_2_, and *j* are the parameters to be estimated, and *Controls*_*it*_ is a series of control variables. *τ*_*i*_ is the provincial individual fixed effect, *μ*_*t*_ is the time-fixed effect, and *ε*_*it*_ is the random disturbance term.

#### Intermediate effect model

To test hypothesis H2, hypothesis H3, the mediation effect model was constructed as follows:

2 $$PMW_{it} = \beta_{0} + \beta_{{1}} New_{it} + \beta_{{2}} New_{it}^{{2}} + jControls_{it} + \tau_{i} + \mu_{t} + \varepsilon_{it}$$

3 $$MCE_{it} = \beta_{0} + \beta_{{1}} New_{it} + \beta_{{2}} New_{it}^{{2}} + \beta_{{3}} PMW_{it} + jControls_{it} + \tau_{i} + \mu_{t} + \varepsilon_{it}$$

4 $$LQ_{it} = \beta_{0} + \beta_{{1}} New_{it} + \beta_{{2}} New_{it}^{{2}} + jControls_{it} + \tau_{i} + \mu_{t} + \varepsilon_{it}$$

5 $$MCE_{it} = \beta_{0} + \beta_{{1}} New_{it} + \beta_{{2}} New_{it}^{{2}} + \beta_{{3}} LQ_{it} + jControls_{it} + \tau_{i} + \mu_{t} + \varepsilon_{it}$$

Among them, *PMW*_*it*_ and *LQ*_*it*_ are mediating variables, representing labour productivity in manufacturing and industrial agglomeration, respectively. Other symbols have the same meaning as above.

### Variable selection

#### Explained variable: manufacturing carbon emissions (MCE)

All existing studies on carbon emissions were obtained by estimation. Referring to the research methods of Ang^33^, Emrouznejad, and Yang^34^, the LMDI model of different industries was used to calculate carbon emissions among 28 sub-industries of China’s manufacturing industry, the specific calculation process is as follows:

Based on Kaya’s constant equation, direct carbon emission coefficient, energy type, energy intensity and other indicators are introduced to extend the constant equation, and the extended Kaya’s constant equation is shown in Eq. (6).

6 $$C = \sum\limits_{i = 1}^{30} {\sum\limits_{j = 1}^{28} {\sum\limits_{k = 1}^{m} {C_{ijk} = \sum\limits_{i,j,k} {E_{ijk} \cdot \varepsilon_{k} \cdot \theta_{j} \cdot Y_{j} \cdot \frac{{Y_{j} }}{Y} \cdot \frac{Y}{P} \cdot P} } } }$$

where: *i* denotes province (30), *j* denotes manufacturing sub-industry (28), *k* denotes energy variety, E_*ijk*_ denotes the *kth* energy consumption of the *jth* industry in province *i*, *ε*_*k*_ denotes the CO_2_ emission coefficient of the *kth* energy source, *θ*_*j*_ denotes the energy intensity of the *jth* industry, *Y*_*j*_ denotes the value-added of the *jth* industry, *Y* denotes the national total manufacturing value-added, and *P* denotes the population size of the province.

#### Core explanatory variable: new quality productivity (New)

This paper mainly refers to the New quality productivity evaluation index system constructed by Lu Jiang et al.^35^ and part of the content proposed by Wang Yu and Wang Rongji^36^, reconstructs and obtains the new quality productivity development index through principal component analysis, and the new quality productivity is recorded as new, the specific evaluation indicators are shown in Table 1.

**Table 1:** Evaluation system for new quality productivity indicators.

Level 1 indicators	Level 2 indicators	Level 3 indicators	Explain	Causality
Technological productivity	Innovative productivity	Innovative research and development	Number of domestic patents granted	+
		Innovation industry	Business income from high-tech industries	+
		Innovative product	Industrial Innovation Funding for Industrial Enterprises Above Scale	+
		Innovation potential	Number of university students as a proportion of the total population	+
	Technological productivity	Technology research and development	The full-time equivalent of R&D personnel in industrial enterprises above the designated size	+
		Technical efficiency	Labour productivity of industrial enterprises on a large scale	+
		Technology to produce	Robot-mounted raw density	+
Green productivity	Resource-efficient productivity	Energy intensity	Energy consumption/ GDP	−
		Energy structure	Fossil energy consumption/GDP	−
		Renewable energy consumption	Renewable energy electricity consumption/electricity consumption of society as a whole	+
	Environmentally friendly productivity	Utilization of waste materials	Comprehensive utilization/generation of industrial solid waste	−
		Wastewater discharge	Industrial wastewater discharges/GDP	−
		Exhaust emission	Industrial SO_2_ emissions/GDP	−
		Environmental protection efforts	Expenditure on environmental protection/ government expenditure on public finance	+
Digital productivity	Digital industry productivity	Electronic Information Manufacturing	IC production	+
		Telecommunications business communications	Total telecommunication services	+
	Industry Digital Productivity	Internet penetration	Number of Internet broadband access ports	+
		Software service	Revenue from software operations	+
		Digital information	Length of fibre optic cable lines/area	+
		E-commerce	E-commerce sales	+

#### Control variable

In this paper, variables such as population density (*Den*), foreign investment level (*For*), income gap between urban and rural residents (*Ingap*), and government intervention capacity (*Govn*) are selected as control variables.

#### Mediating variable: labor productivity in manufacturing (PMW)/Industrial agglomeration (LQ)

The first mediating variable in this paper is labour productivity in manufacturing (denoted by *PMW*). This paper adopts the method of single-factor productivity, which is widely used in academic research, to measure the ratio of the gross manufacturing product to the average annual number of employees. This single-factor productivity measurement method has remarkable rationality and scientificity. In terms of rationality, the GDP of the manufacturing industry intuitively reflects the output results of the industry, the average annual number of employees reflects the scale of input manpower, and the ratio of the two can measure the ability of the unit labour force to create value, which is highly compatible with the concept of labour productivity. From the scientific point of view, the method makes it easy to obtain data, the statistical calibre is clear, and it is convenient to compare the efficiency of the manufacturing industry in different regions and different periods horizontally, it has been widely used in academic researches, and after a large number of empirical tests, the stability and reliability of its results have been verified, and it is able to capture the trend of changes in the manufacturing industry’s labour productivity effectively, and it can provide the subsequent researches with solid data support and analytical basis.

The second mediating variable in this paper is industrial agglomeration (denoted by *LQ*). Combined with the characteristics of the manufacturing industry, the location entropy index not only focuses on geospatial agglomeration but also can better reflect the degree of specialization of the regional measurement industry, which is in line with the measurement index for measuring the level of manufacturing industry agglomeration in each province in China, so the measurement method of this paper is the location entropy method. When the location entropy is greater than 1, it indicates that the manufacturing industry in the province has a high degree of agglomeration; when the location entropy is less than 1, it indicates that the agglomeration level in the province is low. The calculation formula of location entropy is shown in model (7):

7 $$LQ = \sum\limits_{i} {\sum\limits_{t} {\frac{{\frac{{w_{it} }}{{g_{it} }}}}{{\frac{{W_{it} }}{{G_{it} }}}}} }$$

where: *LQ*_*it*_ denotes the industrial agglomeration level of province *i* in year *t*; *w*_*t*_ is the total manufacturing output value of Chinese provinces in year *t*, and *w*_*t*_ is the total manufacturing output value of China in year *t*; *g*_*t*_ and *G*_*t*_ denote the gross regional product (GRP) of Chinese provinces and the gross domestic product (GDP) in year *t*, respectively.

### Sample specification

This paper uses the panel data of 30 provinces in China (excluding Tibet and Hong Kong, Macao, and Taiwan) from 2012 to 2022 as samples. Because some samples did not meet the research requirements, the samples were processed as follows: (1) For a small number of missing values in the samples, the linear interpolation method, near annual mean method, and other methods were used to supplement; (2) Indentation of 1% and 99% of the data was carried out. Data on manufacturing carbon emissions from the China Emission Accounts and Datasets (CEADs); The data on new quality productivity are from the China Energy Statistics Yearbook, China Statistical Yearbook, China Environmental Statistical Yearbook, China Science and Technology Statistical Yearbook, EPS database, statistical yearbooks and statistical bulletins of each province; the data of mediating and control variables are from China Labor Statistical Yearbook, China Statistical Yearbook, China Stock Market Accounting Research Database (CSMAR), provincial statistical yearbooks and bulletins.

The specific variable definitions are shown in Table 2.

**Table 2:** Definition specification.

Type	Name	Notation	Description of definitions
Explained variables	Manufacturing carbon emissions	*MCE*	Calculation of Carbon Emissions by Manufacturing Industry Using the LMDI Model by Sector
Explanatory variables	New quality productivity	*New*	Construct a comprehensive evaluation index system for new quality productivity from the three dimensions of scientific and technological productivity, green productivity and digital productivity, respectively, and use principal component analysis to measure the
Intermediary variable	Labor productivity in manufacturing	*PMW*	The ratio of gross manufacturing product to the average annual number of persons employed
	Industrial agglomeration	*LQ*	Calculated using locational entropy
Control variable	Level of foreign investment	*For*	Expressed in logarithmic terms by the actual amount of foreign investment in China at the end of the year, in which the foreign exchange rate is converted according to the exchange rate of the RMB against the US dollar in that year
	Population density	*Den*	Measured using the percentage of urban population in each province
	Income gap between urban and rural residents	*Ingap*	Tyrell’s index
	Government intervention capacity	*Govn*	Fiscal expenditure as a share of GDP
	Individual effect	*τ*_*i*_	Provincial Individual Fixed Effects
	Year effect	*μ*_*t*_	Year fixed effects

## Empirical result analysis

### Descriptive statistics

The descriptive statistical results of variables are shown in Table 3. It can be seen that the median carbon emissions of the manufacturing industry in the explained variable is 8.2531, the mean is 9.2249, and the variance is 5.2883, indicating that the carbon emission level of the manufacturing industry in the sample varies greatly. The minimum value, maximum value, variance, and mean value of the explanatory variables were 2.8813, 58.5914, 5.6913, and 7.2808, indicating that the new quality productivity as a whole was in the lower middle level.

**Table 3:** Changes descriptive statistical results of variables.

Variable	Sample size	Average value	Medium number	Variance	Minimum value	Maximum value
*MCE*	330	9.2249	8.2531	5.2883	2.4761	12.2793
*New*	330	7.2808	5.8039	5.6931	2.8813	58.5914
*PMW*	330	28.2173	32.4529	27.8475	17.8541	48.6984
*LQ*	330	2.1296	2.3147	1.5247	0.0147	6.2147
*For*	330	0.0183	0.0164	0.0144	0.0001	0.0796
*Den*	330	475.1375	292.8965	707.9262	7.9053	3925.8700
*Ingap*	330	0.0845	0.0800	0.0373	0.0180	0.1970
*Govn*	330	0.2508	0.2281	0.1026	0.1066	0.6430

### What-if analysis

#### Analysis of new and MCE benchmark regression results

The results in column (1) of Table 4 show that the coefficients of *New* and *New*^2^ are both significant at the 1% level, and the coefficients of the primary and secondary terms of New are 3.271 and − 0.246, respectively, indicating an inverted U-shaped relationship between new quality productivity and carbon emissions in the manufacturing industry, and hypothesis H1 has been preliminarily verified.

**Table 4:** Baseline regression and intermediate effect regression results.

Variable	*MCE*	*MCE*	*PMW*	*MCE*	*LQ*
	(1)	(2)	(3)	(4)	(5)
*New*	3.271*** (4.52)	2.156*** (6.38)	-3.169*** (− 8.26)	1.367*** (5.62)	− 0.986*** (− 4.49)
*New* ^2^	− 0.246*** (− 3.10)	− 0.263*** (− 4.13)	0.381*** (6.23)	− 0.193*** (− 2.97)	0.261*** (3.89)
*PMW*		− 0.648*** (− 7.82)			
*LQ*				− 2.153*** (− 7.54)	
_cons	− 8.29 (− 0.35)	3.27*** (3.73)	− 2.68*** (− 4.23)	− 3.19* (− 1.67)	1.52 (0.21)
N	330	330	330	330	330
*Controls*	YES	YES	YES	YES	YES
*τ*_*i*_ * /μ*_*t*_	YES	YES	YES	YES	YES
Adj-R^2^	0.959	0.821	0.939	0.843	0.927

Note: ***, **, and * represent the significance level of 1%, 5%, and 10% respectively, and the t value is in parentheses, the same as below.

#### Analysis of the mediating role of PMW

The results in column (2) of Table 4 show that there is a significant negative correlation between labour productivity in manufacturing and manufacturing carbon emissions and a significant positive correlation between the primary term of new quality productivity and manufacturing carbon emissions. In column (3) of Table 4, the coefficient of the primary term of labour productivity in manufacturing and new quality productivity is significantly negative, which indicates that labour productivity in manufacturing plays a partial mediating role in the influence of new quality productivity on manufacturing carbon emissions. In addition, the mediating effect of labour productivity in manufacturing is 2.053512 (− 0.648 × − 3.169), which accounts for 62.78% of the total effect, verifying the H2 hypothesis.

In this paper, the Bootstrap test is used to determine the robustness of the above mediation effect test results, Table 5, row (1) selects the mediation effect Bootstrap test results for 5000 iterations. The results show that both the direct and indirect effect coefficients are significant and none of the confidence intervals contain zero. Once again, it shows that there is a partial mediating effect of labour productivity in manufacturing in the impact of new quality productivity on carbon emissions in manufacturing, indicating that the results of the analysis of the above mediating effect are robust.

**Table 5:** Bootstrap test result.

Variable	Effect phase	Coefficient	Standard error	P >|Z|	95% confidence interval	
					Lower limit	Upper limit
*PMW*(1)	Direct effect	− 6.859	0.235	0.000	− 8.681	− 5.462
	Indirect effect	− 4.361	0.427	0.000	− 7.191	− 3.286
*LQ*(2)	Direct effect	− 0.368	0.549	0.000	− 0.464	− 0.289
	Indirect effect	− 0.314	0.286	0.000	− 0.485	− 0.217

#### Analysis of the mediating role of LQ

The results in column (4) of Table 4 show that there is a significant negative correlation between industrial agglomeration and carbon emissions from the manufacturing industry and a significant positive correlation between the primary term of new quality productivity and carbon emissions from the manufacturing industry. In column (5) of Table 4, the coefficient of the primary term of industrial agglomeration and new quality productivity is significantly negative, indicating that industrial agglomeration plays a partly intermediary role in the process of the influence of new quality productivity on the carbon emission of the manufacturing industry. The mediating effect of industrial agglomeration is 2.122858 (− 2.153 × − 0.986), which accounts for 64.90% of the total effect, verifying the H3 hypothesis.

The Bootstrap test is still used to determine the robustness of the above-mediated affect test results, the specific results are shown in Table 5, paragraph (2) behavioural choice of 1000 iterations of the mediated effect Bootstrap test results. The results show that the indirect effect coefficients are significant and none of the confidence intervals contain zero. Once again, it shows that there is a partial mediating effect of industrial agglomeration in the impact of new quality productivity on carbon emissions in the manufacturing industry, indicating that the analytical results of the above mediating effect are robust.

### Robustness test

This section conducts robustness tests using three approaches: the Utest method, lagging the explanatory variable by one period, and replacing the explained variable.

The Utest test of hypothesis H1 was carried out by referring to the practice of Lind and Mehlum^37^. The results showed that the extreme point was 6.648, and it was within the value range of the new quality productivity [2.8813, 58.5914]. It follows that the extreme point is within the data range and that the result rejects the null hypothesis at the 1% significance level. In addition, the slope interval is [− 25.556, 1.853] and contains negative values in the interval, which indicates that the influence of new quality productivity on carbon emissions of the manufacturing industry is indeed inverted “U” shape, and the test result of H1 is assumed to be robust.

This paper referred to Bellemare et al.^38^ to delay the primary and secondary terms of the new quality productivity by one stage, and conducted a regression test again. The results are shown in column (1) of Table 6. It can be seen from the test results that the inverted U-shaped relationship between new quality productivity and carbon emissions of the manufacturing industry is robust.

**Table 6:** Robustness test.

Variable	*MCE*	*AMCE*
	(1)	(2)
*L*1*.New*	2.281*** (8.24)	
*L1.New* ^2^	− 3.597*** (− 5.82)	
*New*		0817** (2.53)
*New* ^2^		-0.347*** (-3.61)
_cons	− 2.18 (− 0.13)	3.31*** (8.57)
N	330	330
*Controls*	YES	YES
*τ*_*i*_ * /μ*_*t*_	YES	YES
Adj-R^2^	0.871	0.957

This paper referred to the practice of Li and Lin^39^ and selected per capita carbon emission as the replacement explained variable, expressed by AMCE. The results are shown in column (2) of Table 6. Hypothesis H1 is still valid.

### Endogeneity test

The existence of endogeneity problems may stem from bidirectional causality or omitted variable interference^40^. In this paper, considering that there may be many factors other than new quality productivity affecting carbon emission in the manufacturing industry, there may be the problem of missing variables in the baseline regression results, which may affect the accuracy of the regression results. For this reason, the third power of the difference between the development level of new quality productivity in this province and the mean value of new quality productivity in the rest of the provinces in the same year is chosen as an instrumental variable (denoted by IV). From a correlation perspective, this instrumental variable is highly correlated with the endogenous explanatory variable, new quality productivity, because of the spatial dependence of new quality productivity across provinces, and the structure of the difference captures the relative regional differences: the rest of the provincial averages represent common macro-trends, such as national policies or economic cycles, whose cubic differences from the level of their own province amplify the nonlinear correlation and strengthen the predictive power of the endogenous variable. From an exogeneity perspective, the instrumental variable satisfies the exclusivity condition because the remaining provincial means serve as the reference frame, reflecting exogenous macro factors rather than province-specific disturbances, such as local environmental regulations or stochastic shocks, etc., and the cubic of the difference further isolates the correlation paths and ensures that the instrumental variable affects carbon emissions only indirectly, through the new quality of productivity, and is not related to the error term. Thus, the instrumental variable ensures the robustness and consistency of the regression analysis while mitigating omitted variable bias. Table 7 presents the 2SLS regression results. In the first stage, the instrumental variables are regressed on the quadratic and primary terms of new quality productivity, and it can be seen that the instrumental variable is positively correlated with new quality productivity, which is in line with the real situation. In the second stage, the fitted values of the primary and quadratic terms of new quality productivity are regressed on the carbon emissions of the manufacturing industry, and the inverted U-shape relationship still holds after controlling for endogeneity, which is in line with the previous conclusions. In addition, this paper further conducts a correlation test on the instrumental variables method and finds that the instrumental variables do not have the problem of weak instrumental variables, indicating that the results of this paper are credible.

**Table 7:** Regression results based on 2SLS endogeneity test.

Variable	*New*	*New* ^2^	*MCE*
	First	First	Second
	(4)	(5)	(6)
*IV*	6.4816*** (23.85)	11.3648*** (37.92)	
*New*			6.2539*** (16.74)
*New* ^2^			− 0.3847*** (− 10.82)
_cons	6.8229*** (14.37)	10.6825*** (13.71)	5.1217*** (9.38)
Wald test (Chi-square test /p-value)	16.21 (0.0028)		
N	330	330	330
*Controls*	YES	YES	YES
*τ*_*i*_ * /μ*_*t*_	YES	YES	YES
Adj-R^2^			0.962

### Heterogeneity test

#### Heterogeneity analysis at the level of economic development

According to the practice of Zhang et al.^41^, starting from the level of economic development, eight provinces and cities, including Beijing, Tianjin, Shanghai, Shandong, Guangdong, Jiangsu, Zhejiang, and Fujian, were selected as samples of economically developed provinces and cities, and the rest were samples of economically underdeveloped provinces and cities. As shown in columns (1) and (2) of Table 8, the relationship between new quality productivity and carbon emissions of the manufacturing industry is significantly inverted U-shaped only in economically developed provinces and urban areas, while the relationship between new quality productivity and carbon emissions of the manufacturing industry in economically underdeveloped regions is not inverted U-shaped and is not significant. The reason may be that economically developed provinces and cities usually have more advanced technology and management levels, and the improvement of new quality productivity can bring significant production efficiency improvement in the initial stage, thus increasing the carbon emissions of the manufacturing industry. However, with the continuous improvement of new quality productivity, and the continuous progress of advanced technology, the continuous optimization of transportation management, the manufacturing industry can adapt more quickly and adopt emission reduction measures, and ultimately reduce carbon emissions. However, in economically underdeveloped provinces, the technical level and infrastructure in these areas are relatively backward^42^. New quality productivity is unlikely to have a significant impact on carbon emissions of the manufacturing industry in the region.

**Table 8:** Heterogeneity test.

Variable	Economically developed provinces, cities and districts (1)	Economically underdeveloped provinces, cities and districts (2)	High urbanization level (3)	Low urbanization level (4)
*New*	5.181*** (3.85)	− 1.564 (− 0.80)	2.854** (2.46)	− 1.556 (− 0.81)
*New* ^2^	− 1.072*** (-3.96)	1.469 (0.38)	− 0.758*** (3.97)	1.377 (1.42)
_cons	1.73 (0.97)	− 11.26 (− 0.16)	2.85 (0.14)	− 1.81 (− 0.92)
N	88	242	99	231
*Controls*	YES	YES	YES	YES
*τ*_*i*_ * /μ*_*t*_	YES	YES	YES	YES
Adj-R^2^	0.962	0.833	0.917	0.759

#### Heterogeneity analysis at the level of urbanization

From the perspective of urbanization level, referring to the study of Zhang et al.^43^, the urbanization level is divided into high and low urbanization levels to explore the impact of different urbanization levels on the relationship between new quality productivity and manufacturing carbon emissions. The regression results are shown in columns (3) and (4) of Table 8, and the results show that only in the provinces and municipalities with high urbanization levels, the relationship between new quality productivity and manufacturing carbon emissions is significantly inverted “U”, while in the provinces and municipalities with low urbanization levels, the relationship between new quality productivity and manufacturing carbon emissions does not show an inverted “U” and is not significant. In provinces and cities with low urbanization levels, the relationship between new quality productivity and manufacturing carbon emissions does not show an inverted “U” shape and is not significant. This may be because provinces and cities with a high level of urbanization usually have more advanced technology and management, and the increase of new productivity can bring significant productivity improvement at the initial stage, thus increasing carbon emissions from the manufacturing industry. However, with the continuous improvement of the new quality productivity, which leads to the advancement of advanced technology and the continuous optimization of transportation management, the manufacturing industry can adapt more quickly and take measures to reduce emissions, which ultimately leads to a decrease in carbon emissions. In provinces with a low level of urbanization, the level of technology and infrastructure in these regions is relatively lagging. It is difficult for the new quality productivity to have a significant impact on the carbon emissions of the manufacturing industry in the region.

### Further analysis

To verify the significant difference between the left and right sides of the inverted U-shaped relationship between new quality productivity and carbon emissions from the manufacturing sector, this study adopts the Bootstrap threshold analysis to examine the marginal effects on both sides of the inflexion point of the inverted U-shaped relationship. The regression results are shown in columns (1) and (2) of Table 9. Based on the previous U-test test, the value of the inflexion point of the inverted U-shaped curve is obtained as 6.648, 5.000 repetitive samples were taken to construct the confidence intervals of the inflexion point by nonparametric Bootstrap, and the results show that the estimated value of the inflexion point is significantly deviated from the endpoint of the sample at the 1% level (Wald’s test χ^2^ = 18.37, p < 0.01), which indicates that the inverted U-shaped relationship is statistically significant. Further segmented regression tests on both sides of the inflexion point found that when the new quality productivity is lower than 6.648, its regression coefficient is 1.326, that is, the new quality productivity enhancement will significantly exacerbate the carbon emissions of the manufacturing industry; and when the new quality productivity crosses the inflexion point, the regression coefficient turns to − 0.814, which indicates that the progress of the new quality productivity has a significant inhibition of the emission reduction of the manufacturing industry. Bootstrap sub-sample test shows that the F-statistic of the difference in coefficients on both sides is 23.19 (p = 0.000), rejecting the original hypothesis of equal slopes. This finding verifies the stage characteristic of the environmental Kuznets curve, i.e., there exists a critical threshold from quantitative to qualitative changes in the impact of new quality productivity on carbon emissions, and the synergistic effect of labour productivity in manufacturing and green transformation can only be released when the productivity breaks through the threshold value of 6.648.

**Table 9:** Results of the two-sided effects test for the inverted U-shaped relationship.

Variable	Left side of the inflection point (New < 6.648) (1)	Right side of the inflection point (New > 6.648) (2)	Difference test (P-value)
*New*	1.326** (0.514)	-0.814*** (0.076)	0.000
*Controls*	YES	YES	
N	102	228	
Adj-R^2^	0.779	0.968	

Note: Bootstrap standard errors are in parentheses.

## Discussion and conclusion

Based on the panel data of 30 provinces in China (excluding Tibet, Hong Kong, Macao, and Taiwan) from 2012 to 2022, the mechanism of the impact of new quality productivity on carbon emissions of the manufacturing industry was empirically investigated. The following conclusions are obtained:

1. There is an inverted U-shaped relationship between new quality productivity and carbon emissions of the manufacturing industry, that is, with the improvement of new quality productivity, carbon emissions of the manufacturing industry first increase and then decrease. Therefore, the manufacturing industry should fully understand the “two sides” of the after-effects of improving the new quality productivity and continue to develop the new quality productivity of logistics. Although the process of improving logistics and new quality productivity will lead to an increase in carbon emissions in the manufacturing industry, in the long run, the improvement of new quality productivity is conducive to the reduction of carbon emissions in the manufacturing industry. Therefore, the manufacturing industry should vigorously develop new quality productivity, actively carry out technological innovation, and apply the innovation results to specific manufacturing businesses to achieve the upgrade and leap of logistics “productivity”. Accelerate the green transformation of the manufacturing industry and help the manufacturing industry reduce carbon emissions.
2. Labor productivity in manufacturing and industrial agglomeration plays a partial mediating role in the relationship between new quality productivity and carbon emissions from manufacturing. Moreover, the mediating effect of industrial agglomeration is stronger than that of labour productivity in manufacturing. Therefore, the manufacturing industry should insist on multiple measures to accelerate industrial agglomeration and enhance labour productivity in manufacturing. On the one hand, in terms of industrial agglomeration, scientific planning of industrial park layout, based on the resource endowment and industrial foundation of different regions, to create manufacturing industry clusters with distinctive features and complementary advantages^44^. Improve the infrastructure construction of industrial parks, including energy supply, waste treatment, etc., to provide good hardware support for industrial clustering and improve the efficiency of industrial synergy. Strengthen the cooperation and communication between enterprises in industrial clusters, build a symbiotic relationship of close collaboration between the upstream and downstream of the industrial chain, realize resource sharing, technology complementation and waste recycling, and form a green and low-carbon industrial ecosystem. On the other hand, In terms of labour productivity in the manufacturing industry, manufacturing enterprises need to promote intelligent technology and production system synergistic upgrading, through the industrial Internet, artificial intelligence and the depth of the application of advanced automation equipment, reconfiguration of the production process and process paradigm, breakthrough of the traditional bottleneck of efficiency, to achieve output per unit of time to jump up. Moreover, institutions of higher education should strengthen the supply of high-quality labour, build a skills training system that is compatible with intelligent manufacturing, focus on cultivating composite skills such as digital operation and maintenance and green process optimization, and improve the incentive mechanism to stimulate the effectiveness of human capital. In addition, it is also crucial to optimize the structure of factor allocation, promote the flow of capital from energy-consuming links to high-efficiency areas, and support manufacturing enterprises to reduce production redundancy through the intelligent updating of equipment and lean management mode.
3. The impact of new quality productivity on carbon emissions from manufacturing industries varies among different levels of regional economic development, and the impact of new quality productivity on carbon emissions from manufacturing industries in economically developed and highly urbanized provinces and cities is more obvious than that in economically less developed and low-urbanized provinces and cities. Therefore, the government should adopt comprehensive measures to enhance the development level of new quality productivity in economically underdeveloped and low-urbanized regions in China. The government should set up a special scientific research fund to encourage enterprises and scientific research institutions to carry out technological research and development for energy saving and emission reduction in the manufacturing industry.^45^ At the same time, preferential policies should be introduced to attract high-end talents to move to the less developed and low-urbanized areas, to provide intellectual support for the development of new quality productivity. By upgrading the level of science and technology and the reserve of talent, the manufacturing industry in economically underdeveloped and low-urbanized areas will be promoted to develop in the direction of high efficiency and low carbon^[46,^^47]^.

## Data Availability

The data are available from the corresponding author upon reasonable request.

## References

[1] Li, W. et al. Spatio-temporal impacts of land use change on water-energy-food nexus carbon emissions in China, 2011–2020. *Environ. Impact Assess. Rev.* **105**, 107436. 10.1016/j.eiar.2024.107436 (2024).

[2] Xiao, W. Viewing the development of new - quality productivity from the perspective of Marxism. *Ideol. Theor. Educ.* **4**, 12–19. 10.16075/j.cnki.cn31-1220/g4.2024.04.004 (2024).

[3] Xu, Z., Zhang, J. Y. & Li, Z. Y. New - quality productivity empowering carbon peak and carbon neutrality: intrinsic logic and practical strategies. *Qinghai Soc. Sci.* **6**, 30–39. 10.14154/j.cnki.qss.2023.06.003 (2023).

[4] Wang, L., Chen, Q., Dong, Z. & Cheng, L. The role of industrial intelligence in peaking carbon emissions in China. *Technol. Forecast. Soc. Chang.* **199**, 123005. 10.1016/j.techfore.2023.123005 (2024).

[5] Branca, T. A. et al. Industrial symbiosis and energy efficiency in European process Industries: A review. *Sustainability* **13**(16), 9159. 10.3390/su13169159 (2021).

[6] Wang, S. & Chen, F. Can new quality productivity promote the carbon emission performance—empirical evidence from China. *Sustainability* **17**(2), 567. 10.3390/su17020567 (2025).

[7] Liu, D., Zhu, X. & Wang, Y. China’s agricultural green total factor productivity based on carbon emission: an analysis of evolution trend and influencing factors. *J. Clean. Prod.* **278**, 123692. 10.1016/j.jclepro.2020.123692 (2021).

[8] Fu, M., Xu, Z. & Ge, L. M. The intrinsic logic and practical paths of new - quality productivity boosting the rise of Central China. *J. Zhengzhou Univ.* **57**(5), 70–76 (2024).

[9] Lin, L., Gu, T. & Shi, Y. The influence of new quality productive forces on high-quality agricultural development in China: mechanisms and empirical testing. *Agriculture* **14**(7), 1022. 10.3390/agriculture14071022 (2024).

[10] Zhang, J. et al. Study on the coordinated development degree of new quality productivity and manufacturing carbon emission efficiency in provincial regions of China. *Environ. Dev. Sustain.* **2024**, 1–35. 10.1007/s10668-024-05321-x (2024).

[11] Li, X. & Yue, S. Blessing or curse? The role of digital technology innovation in carbon emission efficiency. *J. Environ. Manage.* **365**, 121579. 10.1016/j.jenvman.2024.121579 (2024). 38936018 10.1016/j.jenvman.2024.121579

[12] Yu, Z., Liu, Y., Yan, T. & Zhang, M. Carbon emission efficiency in the age of digital economy: new insights on green technology progress and industrial structure distortion. *Bus. Strateg. Environ.* **33**(5), 4039–4057. 10.1002/bse.3683 (2024).

[13] Liang, L., Huang, C. & Hu, Z. Industrial structure optimization, population agglomeration, and carbon emissions—empirical evidence from 30 provinces in China. *Front. Environ. Sci.* **10**, 1078319. 10.3389/fenvs.2022.1078319 (2023).

[14] Liu, J., Cheng, Z. & Zhang, H. Does industrial agglomeration promote the increase of energy efficiency in China?. *J. Clean. Prod.* **164**, 30–37. 10.1016/j.jclepro.2017.06.179 (2017).

[15] Ahn, Y. J. & Juraev, Z. Critical analysis of Marxist ideas in modern urban planning. *Cities* **148**, 104843. 10.1016/j.cities.2024.104843 (2024).

[16] Wang, J., Qiao, L., Zhu, G., Di, K. & Zhang, X. Research on the driving factors and impact mechanisms of green new quality productive forces in high-tech retail enterprises under China’s Dual Carbon Goals. *J. Retail. Consum. Serv.* **82**, 104092. 10.1016/j.jretconser.2024.104092 (2025).

[17] Qian, L., Jin, Y. T. & Ma, C. Y. Digital new quality productivity and increasing farmers’ income: theoretical analysis and empirical evidence. *J. Southwest Univ.* **50**(05), 15–30. 10.13718/j.cnki.xdsk.2024.05.002 (2024).

[18] Masanet, E., Shehabi, A. & Lei, N. Recalibrating global data center energy-use estimates. *Science* **367**(6481), 984–986. 10.1126/science.aba3758 (2020). 32108103 10.1126/science.aba3758

[19] Qi, K., Owusu, E. K., Siu, M. F. F. & Chan, P. C. A. A systematic review of construction labor productivity studies: clustering and analysis through hierarchical latent dirichlet allocation. *Ain Shams Eng. J.* **2024**, 102896. 10.1016/j.asej.2024.102896 (2024).

[20] Wu, H., Wen, H., Li, G., Yin, Y. & Zhang, S. Unlocking a greener future: the role of digital finance in enhancing green total factor energy efficiency. *J. Environ. Manage.* **364**, 121456. 10.1016/j.jenvman.2024.121456 (2024). 38875989 10.1016/j.jenvman.2024.121456

[21] Shrouf, F. & Miragliotta, G. Energy management based on Internet of Things: practices and framework for adoption in production management. *J. Clean. Prod.* **100**, 235–246. 10.1016/j.jclepro.2015.03.055 (2015).

[22] Wang, C. A., Liu, X., Li, H. & Yang, C. Analyzing the impact of low-carbon city pilot policy on enterprises’ labor demand: evidence from China. *Energy Econ.* **124**, 106676. 10.1016/j.eneco.2023.106676 (2023).

[23] Yang, T. & Lai, S. Redefine manufacturing operations for modern production environments with the help of artificial intelligence enterprise information systems. *Int. J. Adv. Manufact. Technol.* **2024**, 1–12. 10.1007/s00170-024-14838-4 (2024).

[24] Fareri, S., Apreda, R., Mulas, V. & Alonso, R. The worker profiler: assessing the digital skill gaps for enhancing energy efficiency in manufacturing. *Technol. Forecast. Soc. Chang.* **196**, 122844. 10.1016/j.techfore.2023.122844 (2023).

[25] Krupas, M., Kajati, E., Liu, C. & Zolotova, I. Towards a human-centric digital twin for human–machine collaboration: a review on enabling technologies and methods. *Sensors* **24**(7), 2232. 10.3390/s24072232 (2024). 38610442 10.3390/s24072232 PMC11013982

[26] O’Donoghue, D. & Gleave, B. A note on methods for measuring industrial agglomeration. *Reg. Stud.* **38**(4), 419–427. 10.1080/03434002000213932 (2004).

[27] Wang, B., Sun, Y. & Wang, Z. Agglomeration effect of CO_2_ emissions and emissions reduction effect of technology: a spatial econometric perspective based on China’s province-level data. *J. Clean. Prod.* **204**, 96–106. 10.1016/j.jclepro.2018.08.243 (2018).

[28] Mangone, G. Constructing hybrid infrastructure: Exploring the potential ecological, social, and economic benefits of integrating municipal infrastructure into constructed environments. *Cities* **55**, 165–179. 10.1016/j.cities.2016.04.004 (2016).

[29] Liu, X. & Zhang, X. Industrial agglomeration, technological innovation and carbon productivity: evidence from China. *Resour. Conserv. Recycl.* **166**, 105330. 10.1016/j.resconrec.2020.105330 (2021).

[30] Ye, J., Wan, Q., Li, R., Yao, Z. & Huang, D. How do R&D agglomeration and economic policy uncertainty affect the innovative performance of Chinese high-tech industry?. *Technol. Soc.* **69**, 101957. 10.1016/j.techsoc.2022.101957 (2022).

[31] Karaev, A., Lenny Koh, S. C. & Szamosi, L. T. The cluster approach and SME competitiveness: a review. *J. Manuf. Technol. Manag.* **18**(7), 818–835. 10.1108/17410380710817273 (2007).

[32] Lu, H. P. & Weng, C. I. Smart manufacturing technology, market maturity analysis and technology roadmap in the computer and electronic product manufacturing industry. *Technol. Forecast. Soc. Chang.* **133**, 85–94. 10.1016/j.techfore.2018.03.005 (2018).

[33] Ang, B. W. Decomposition analysis for policymaking in energy: which is the preferred method?. *Energy Policy* **32**(9), 1131–1139. 10.1016/s0301-4215(03)00076-4 (2004).

[34] Emrouznejad, A. & Yang, G. L. A framework for measuring global Malmquist-Luenberger productivity index with CO2 emissions on Chinese manufacturing industries. *Energy* **115**, 840–856. 10.1016/j.energy.2016.09.032 (2016).

[35] Lu, J., Guo, Z. A. & Wang, Y. P. Levels of development of new quality productivity, regional differences and paths to enhancement. *J. Chongqing Univ.* **30**(3), 1–17. 10.11835/j.issn.1008-5831.jg.2024.03.002 (2024).

[36] Wang, Y. & Wang, R. J. New quality productivity: index construction and spatiotemporal evolution. *J. Xi’an Univ. Finance Econ.* **37**(1), 31–47. 10.19331/j.cnki.jxufe.20231124.001 (2024).

[37] Lind, J. T. & Mehlum, H. With or without U? The appropriate test for a U-shaped relationship. *Oxford Bull. Econ. Stat.* **72**(1), 109–118. 10.1111/j.1468-0084.2009.00569.x (2010).

[38] Bellemare, M. F., Masaki, T. & Pepinsky, T. B. Lagged explanatory variables and the estimation of causal effect. *J. Politics* **79**(3), 949–963. 10.1086/690946 (2017).

[39] Li, X. & Lin, B. Global convergence in per capita CO2 emissions. *Renew. Sustain. Energy Rev.* **24**, 357–363. 10.1016/j.rser.2013.03.048 (2013).

[40] Zaefarian, G., Kadile, V., Henneberg, S. C. & Leischnig, A. Endogeneity bias in marketing research: problem, causes and remedies. *Ind. Mark. Manage.* **65**, 39–46. 10.1016/j.indmarman.2017.05.006 (2017).

[41] Zhang, X., Li, C., Li, W., Song, J. & Yang, C. Do administrative boundaries matter for uneven economic development? A case study of China’s provincial border counties. *Growth Chang.* **48**(4), 883–908. 10.1111/grow.12196 (2017).

[42] Li, H., Zhang, Y. & Li, Y. The impact of the digital economy on the total factor productivity of manufacturing firms: empirical evidence from China. *Technol. Forecast. Soc. Chang.* **207**, 123604. 10.1016/j.techfore.2024.123604 (2024).

[43] Zhang, B., Zhang, J. & Miao, C. Urbanization level in Chinese counties: imbalance pattern and driving force. *Remote Sens.* **14**(9), 2268. 10.3390/rs14092268 (2022).

[44] Liu, Y. & He, Z. Synergistic industrial agglomeration, new quality productive forces and high-quality development of the manufacturing industry. *Int. Rev. Econ. Financ.* **94**, 103373. 10.1016/j.iref.2024.103373 (2024).

[45] Zhou, Y. & Lin, B. The energy-saving effect of green fiscal policy: empirical evidence from China’s comprehensive demonstration cities of energy conservation and emission reduction fiscal policy. *Appl. Energy* **378**, 124784. 10.1016/j.apenergy.2024.124784 (2025).

[46] Xu, F. & Xu, H. Evaluation of the Degree of Coordination between Provincial Ecological Protection and High-Quality Development andDiagnosis of Obstacle Factors. *Pol. J. Environ. Stud.* **32**, (1), 371–385. 10.15244/pjoes/152985(2023).

[47] Xu, F., Xu, H. & Yan, F. Spatial-Temporal Characteristics and Obstacle Factors of Industry Ecology in the Lower Yellow River. *Pol. J. Environ. Stud.* **32**, (1), 901–912. 10.15244/pjoes/155146(2023).

